# *Aeromonas veronii* Is a Lethal Pathogen Isolated from Gut of Infected *Labeo rohita*: Molecular Insight to Understand the Bacterial Virulence and Its Induced Host Immunity

**DOI:** 10.3390/pathogens12040598

**Published:** 2023-04-14

**Authors:** Bijay Kumar Behera, Satya Narayan Parida, Vikash Kumar, Himanshu Sekhar Swain, Pranaya Kumar Parida, Kampan Bisai, Souvik Dhar, Basanta Kumar Das

**Affiliations:** 1 Aquatic Environmental Biotechnology and Nanotechnology Division, ICAR-Central Inland Fisheries Research Institute, Kolkata 700120, West Bengal, India; satya.parida4@gmail.com (S.N.P.); kumar.vika.vikash2@gmail.com (V.K.); pranayaparida@gmail.com (P.K.P.); kampanbisai@gmail.com (K.B.); dharsouik2014@gmail.com (S.D.); 2 Aquaculture Production and Environment Division, ICAR-Central Institute of Freshwater Aquaculture, Bhubaneswar 751002, Orissa, India; himanshufishco@gmail.com

**Keywords:** mortality, 16S rRNA gene, virulence gene, immunity

## Abstract

A case of severe mortality in farmed *Labeo rohita* was investigated to characterize the causative agent. We identified the bacterial strain as *Aeromonas veronii* isolated from the gut of infected *L. rohita* by biochemical assay, scanning electron microscopy and 16S rRNA gene sequence analysis. The in vivo challenge experiment showed that the LD_50_ of *A. veronii* was 2.2 × 10^4^ CFU/fish. Virulence gene investigation revealed that the isolated *A. veronii* possesses Aerolysin, Cytotoxic enterotoxin, Serine protease, Dnase and Type III secretion system genes. The isolated strain was resistant to two antibiotics (ampicillin and dicloxacillin) while susceptible to 22 other antibiotics. The study further revealed that *A. veronii* induced both stresses along with non-specific and specific immune responses marked by elevated cortisol HSP70, HSP90 and IgM levels in the treated *L. rohita* fingerlings. Although the bacterial pathogen enhances the immune response, the negative effect on fish, including stress, and high mortality, create concern and a need for *A. veronii* management in *L. rohita* farms. The knowledge gained from this study would facilitate future research aimed at assessing the pathogenicity of *A. veronii*, with an emphasis on microbial disease management in other farmed fish species.

## 1. Introduction

Fish contribute ~20% of total global animal protein intake, demonstrating its relevance in global food security and nutrition. India is the world’s second-largest aquaculture producer, with Indian Major Carps (IMCs) contributing to more than 70% of the country’s aquaculture sector [1]. The IMC, *Labeo rohita* (Rohu), is one of the most cultured finfish species produced in India and worldwide aquaculture [2,3,4], with a greater preference by consumers [5,6]. The East Medinipur district is considered the hub of aquaculture in West Bengal, India, which accounts for the largest production of Indian Major Carp, including *L. rohita* [1]. However,, due to the global demand increase, the pressure for intensification and further expansion of aquaculture has created many problems, including scarcity of natural resources, increased environmental pollution and losses due to disease outbreaks [1,7,8]. Disease outbreaks caused by microbial pathogens, considered the primary cause of production loss in fish farming, have moved to the forefront in recent years and brought socio-economic and environmental unsustainability to the aquaculture industry [9,10].

Among the various known pathogenic bacteria, *Aeromonas veronii* is a Gram-negative bacterium from the family Aeromonadaceae that has been frequently discovered in water, soil and food [1,7,8,9,10]. *A. veronii* is known for causing pandemics and epidemics in freshwater fishes, resulting in significant economic loss to the aquaculture sector [11,12,13,14]. The clinical signs of *A. veronii* infection include ulceration, abdominal distention, exophthalmia, hemorrhagic septicemia and fin rot [15,16]. As previously reported, *A. veronii* has been isolated from several fish species, *Micropterus salmoides* [17], *Carassius auratus gibelio* [18], *Anabas testudinues* [19], *Leiocassis longirostris gunther* [20], *C. auratus*, *Cyprinus carpio*, *Ctenopharyngodon idella*, *Silurus asotus* [21], *Channa argus* [22], catfish [23], *Ictalurus punctatus* [24], *Astronotus ocellatus* [25], *Poecilia reticulate* [26] and *Misgurnus anguillicaudatus* [27]. The virulence factors play a pivotal role in the pathogenesis of bacterium in fish species [28,29,30]. Recently a new strain of *A. veronii* was characterized, isolated from diseased *L. rohita* based on the NGS approach revealing the whole genome sequence of the bacteria [31]. However, a systematic study on the *A. veronii* virulence factor, host immunity, and survival has not been addressed yet. Hence, there is a need to understand the mechanism and characteristics this bacterial pathogen to develop a suitable management method and reduce disease outbreaks and mortality in farmed fishes.

The study aimed to characterize a disease condition that indicated the possible involvement of a bacterial pathogen. For this study, the etiological agent responsible for disease outbreaks in Indian major carp, *L. rohita*, was collected from an aquaculture farm situated at East Medinipur, West Bengal, India. Furthermore, the bacterium was characterized through biochemical assay, Scanning Electron Microscopy (SEM), 16S rRNA gene sequencing and phylogenetic analysis. Afterward, the in vivo challenge assay, virulence gene characterization and histological changes were studied to determine the pathogenic potential of isolated *A. veronii* in healthy fingerlings of *L. rohita*. Later, HSP70, HSP90, IgM and cortisol levels were also analyzed to investigate the immune-stress response of challenged *L. rohita*.

## 2. Materials and Methods

### 2.1. Sample Collection

**Figure 1:**
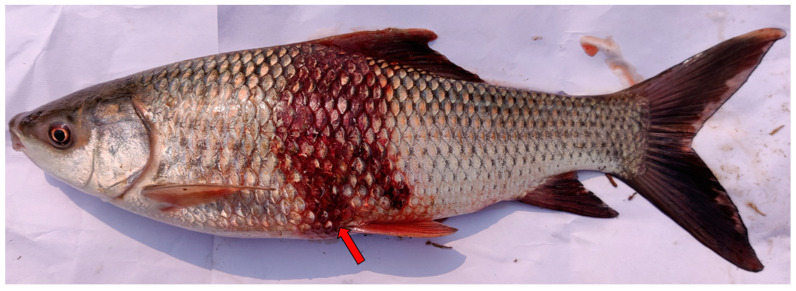
Diseased fish sample of *Labeo rohita* collected from East Medinipur, West Bengal, India, indicating a hemorrhage on the body surface (arrow in red color).

Six diseased and moribund *L. rohita* (Figure 1) were collected from a fish farm of East Medinapur (Latitude 22.107897°, Longitude 87.907583°), West Bengal, India (Figure S1) following the standard protocol [10]. The aquaculture farms have reported about 40% mortality in the total fish population. The fish that showed clinical signs, including redness and hemorrhages on body surfaces, were collected from the aquaculture farm and transferred to a 50 L capacity FRP tank with constant aeration in the fish pathology lab (ICAR-Central Inland Fisheries Research Institute), Kolkata, for screening of etiological agent. Clinical and post-mortem examinations were analyzed according to standard protocol [32]. The animal utilization protocol was approved by Institutional Animal Ethics Committee, ICAR-CIFRI, Kolkata, India (IAEC/2021/04) for the experimental setup.

### 2.2. Bacterial Screening from the Infected Fish

The fish with clinical signs and lesions on the body was sacrificed by using clove oil as anesthetic (Dabur, Ghaziabad, India) at 50 µL per liter of water. The gut samples were collected aseptically and incubated in TSB (HiMedia, Mumbai, India) for 24 h at 28 °C. The overnight sample suspension was diluted to 10^−6^ and spread on TSA (HiMedia, Mumbai, India) plate, and a single colony was streaked on a new fresh TSA plate to acquire a pure culture. The culture from TSB media was also streaked on the *Aeromonas*-specific media with Aeromonas selective supplement (HiMedia, Mumbai, India) and incubated at 28 °C for 24 h. The pure culture of bacteria was then maintained at 30% Glycerol stock and stored at −80 °C.

### 2.3. Biochemical Characterization

The bacterial isolate was primarily distinguished by the Gram-staining technique [33]. The strain was then subjected to biochemical characterization following standard procedure, *viz*., lysine utilization, oxidase, lactose, trehalose, glucose, raffinose, melibiose, saccharose, adonitol, cellobiose, rhamnose, xylose, arabinose, malonate utilization, esculin hydrolysis, indole, Voges Proskauer’s (VP), methyl red, H_2_S production, citrate utilization, phenylalanine utilization, nitrate reduction, urease and ONPG (β-galactosidase) by using the biochemical kit (KB003, HiMedia, Mumbai, India).

### 2.4. SEM Analysis

The bacterial isolate was sub-cultured overnight at 28 °C in 50 mL Erlenmeyer flasks (Himedia, India) containing 20 mL of sterile Tryptone soya broth (TSB). Afterward, 50 µL of bacterial suspension was placed onto a two cm length glass slide, and a smear was prepared. The bacterial smear was air-died for 1 h and 100 µL of 2.5% glutaraldehyde was added onto the top of the smear and incubated at 4 °C for 6 h. Later, the slides were washed with sterile phosphate buffer saline (PBS, HiMedia, Mumbai, India) two to three times [34]. The slide was dehydrated in graded ethanol series (30, 50, 70 and 100% ethanol) for 10 min for each concentration. Subsequently, the slides were placed into a dark glass bottle containing 100% acetone and transferred to the Centre for Research in Nanoscience and Nanotechnology (CRNN) facility, Calcutta University, West Bengal, India, for scanning electron microscopy (SEM) (Evo 18 special edition, Zeiss, Oberkochen, Germany).

### 2.5. Molecular Characterization by 16S rRNA Gene and Phylogeny Analysis

The sarkosyl method was used to isolate the bacterial genomic DNA [35]. The isolated DNA was checked on 1.8% agarose gel, and the quality of the DNA was studied by using Nano-drop (Eppendorf, Hamburg, Germany). The 16S rRNA gene was amplified using 96-well thermal cycler PCR system 9700 (Applied biosystem, Foster City, CA, USA) using the primer listed in Table 1. The final volume of the PCR reaction mixture maintained at 50 µL consists of 1 μL of 10 mM dNTP, 100 ng of isolated genomic DNA, 1 μL of 50 mM MgCl_2_, 1 μL of 10 pmol of each primer, 1 µL of Taq DNA Polymerase and 5 μL of 10× PCR buffer (Sigma, New Haven, CT, USA). The thermal condition was maintained with initial denaturation (2 min at 95 °C), 35 cycles of denaturation (94 °C for 30 s), annealing (52 °C for 60 s), extension (72 °C for 90 s) and final extension (7 min at 72 °C). The PCR products were visualized on 1.8% agarose gel [36]. Using an ABI 373xl capillary sequencer, the amplified gene was sequenced in both directions (Applied Biosystem, Foster City, CA, USA). A contig was created by matching forward and reverse sequences using DNA Baser 7.0 and the sequence was submitted to NCBI, GeneBank. The 16S rRNA gene sequence of MP3 was around 1403 bp, which was then compared with other sequences available in GeneBank using the NCBI-BLAST program. The 16S rRNA gene sequence of MP3 was aligned with 16S rRNA gene sequences of the most identified *Aeromonas* sp. retrieved from the NCBI gene bank. MEGA 11.0 [37] was used to generate a phylogenetic tree using the neighbor-joining method [38] and iTOL v4 (Interactive Tree of Life) [39] software was used to design the phylogenetic tree.

### 2.6. Hemolytic Property

Bacterial hemolytic activity was examined in both solid and liquid phases. The experiment was carried out in the solid phase by streaking the pure bacterial culture on blood agar (5% sheep blood) and incubating for 24 h at 37 °C, and the percentage was calculated according to [40]. Furthermore, the experiment was carried out in the liquid phase using 2% sheep red blood cells. To perform the liquid phase assay, the bacterial cells were centrifuged for 5 min at 5000 rpm, and the resulting cell pellet was washed three times with PBS before being resuspended in PBS. The bacterial cell suspension and Sheep RBC were gently mixed in a 1:1 ratio, with a final 2% RBC concentration maintained [33]. After centrifuging the mixture for 30 min at 1200 rpm, it was incubated at 37 °C. Later, the hemolysis was observed spectrophotometrically at 540 nm [41] every 2 h until 18 h [40] on CLARIOSTAR ^R^ Plus (BMG Labtech, Ortenberg, Germany).

### 2.7. Antibiogram Assay

The in vitro agar diffusion technique was used to evaluate the antibiotic susceptibility of bacterial strains using antibiotic discs (HiMedia, Mumbai, India) [42,43]. The study was conducted by using 24 different antibiotic discs (6 mm diameter) like Ampicillin (AMP 25), Dicloxacillin (D/C 1), Ofloxacin (OF2), Erythromycin (E 10), Gentamicin (GEN 10), Netilmicin sulfate (NET30), Amoxicillin (AMC 30), Tetracycline (TE10), Chloramphenicol (C30), Cefixime (CFM5), Piperacillin (PIT100/10), Nalidixic Acid (NA30), Imipenem (IPM10), Colistin (CL10), Doxycycline, (DO10), Trimethoprim (TR5), Fosfomycin (FO200), Rifampicin (RIF5), Nitrofurantoin (NIT 200), Tobramycin (TOB10), Cefepime (CPM30), Polymyxin B (PB300), Ciprofloxacin (CIP5) and Streptomycin (S25). The overnight-grown bacteria culture was calculated by spread plate method, and 100 µL of pure bacterial culture (5.8 × 10^8^ CFU/mL) was spread on each TSA plate using a plate spreader [44]. Five to six different antibiotic discs were positioned on an individual plate. The agar plates were then rapped with parafilm and incubated for 24 h at 37 °C. The diameters of the inhibitory halos bordering the antibiotic discs were calculated by millimeters following the Clinical and Laboratory Standards Institute [45]. The results were classified as sensitive, intermediate, and resistant.

### 2.8. Identification of Virulence Genes

The virulence genes like aerA (Aerolysin), act (Cytotoxic enterotoxin), ser (Serine protease), gcaT (Glycerophospholipid: cholesterol acyltransferase), Lip (Lipase), ast (Cytotonic enterotoxin), alt (Heat labile cytotonic enterotoxin), ahyB (Elastase), exu (Dnase), hlyA (Hemolysins) and ascV (Type III secretion system) were analyzed individually using gene-specific primers (Table 1). The conventional procedures for DNA extraction by the sarkosyl method, virulence gene amplification, gel electroporation, and gel image were taken according to the process described previously [33].

**Table 1:** Primer list used to amplify the16S rRNA and virulent genes.

S. No.	Primers	Gene	Size	Tm (°C)	Reference
1	UFF2:—GTTGATCATGGCTCAG	16S rRNA	1450	52	[36]
	URF2:—GGTTCACTTGTTACGACTT				
2	aerA-F CCTATGGCCTGAGCGAGAAG	Aerolysin	431	63	[23]
	aerA-R CCAGTTCCAGTCCCACCACT				
3	act-F AGAAGGTGACCACCACCAAGAACA	Cytotoxic enterotoxin	232	65	[23]
	act-R AACTGACATCGGCCTTGAACTC				
4	Ser-F CACCGAAGTATTGGGTCAGG	serine protease	350	57	[23]
	Ser-R GGCTCATGCGTAACTCTGGT				
5	gcaT-F CTCCTGGAATCCCAAGTATCAG	Glycerophospholipid: cholesterol	237	65	[17]
	gcaT-R GGCAGGTTGAACAGCAGTATCT	acyltransferase			
6	Lip-F ATCTTCTCCGACTGGTTCGG	lipase	382	64	[17]
	Lip-R CCGTGCCAGGACTGGGTCTT				
7	ast-F TCTCCATGCTTCCCTTCCACT	Cytotonic enterotoxin	331	63	[17]
	ast-R GTGTAGGGATTGAAGAAGCCG				
8	alt-F TGACCCAGTCCTGGCACGGC	Heat-labile cytotonic enterotoxin	442	64	[23]
	alt-R GGTGATCGATCACCACCAGC				
9	ahyB-F ACACGGTCAAGGAGATCAAC	Elastase	513	59	[23]
	ahyB-R CGCTGGTGTTGGCCAGCAGG				
10	exu-F AGACATGCACAACCTCTTCC	Dnase	323	60	[17]
	exu-R GATTGGTATTGCCTTGCAAG				
11	hlyA F GGCCGGTGGCCCGAAGATACGGG	Hemolysins	597	62	[46]
	hlyA R GGCGGCGCCGGACGAGACGGG				
12	ascV-F AGCAGATGAGTATCGACGG	Type III Secretion System	891	58	[46]
	ascV-R AGGCATTCTCCTGTACCAG				

### 2.9. Pathogenicity Study and LD_50_ Determination

Healthy *L. rohita* of fingerling stage size (length 115.52 ± 2.16 mm and weight 20.26 ± 1.02 g) were procured from the same stock of a nearby hatchery and acclimatized for 14 days in properly aerated conditions by feeding with commercially available fish feed (2% of the fish body weight). The isolated bacteria were subcultured in TSB in a 15 mL culture tube (Abdos, Howrah, India) and incubated at 37 °C for 24 h. The bacterial culture was then centrifuged for 5 min at 5000 rpm, the supernatant was removed, and the pellet was washed twice and resuspended in sterile normal saline [33]. A total of 240 fish were randomly segregated into 24 glass tanks, each with a 150 L water capacity. All the tanks were divided into eight experimental groups (one control and seven treated groups). The bacterial concentration was calculated according to the spread plate method. The fishes of each treated group were injected intraperitoneally with bacteria mix (0.2 mL per fish) with ultimate concentrations of 1.2 × 10^2^, 1.2 × 10^3^, 1.2 × 10^4^, 1.2 × 10^5^, 1.2 × 10^6^, 1.2 × 10^7^ and 1.2 × 10^8^ CFU/fish. Fish in the control group were only injected with 0.2 mL of sterile normal saline. During the experimental trial, the water temperature was between 28–30 °C and other water quality parameters like pH, dissolved oxygen and alkalinity was 7.30 ± 0.05, 5.3 ± 0.30 mg/L and 78.0 ± 1.4 mg/L, respectively. The fish were monitored every 12 h for mortality/morbidity over a period of 7 days. Moreover, mortalities were registered every 24 h until 168 h post-injection. The bacteria were re-isolated from the bacterial-challenged fish by anesthetizing with clove oil (Dabur, Ghaziabad, India) (50 µL per liter of water) to satisfy Koch’s postulate. The calculation of LD_50_ was accomplished by using the standard method described [47].

### 2.10. Collection of Serum Sample

For serum sample analysis, a total of 60 healthy fishes (length 115.52 ± 2.16 mm and weight 20.26 ± 1.02 g) were distributed among six experimental glass tanks, each with 150 L water capacity (three tanks for control and three for the bacteria-challenged group). One-tenth dose of LD_50_ (~2.2 × 10^3^ CFU/fish) was injected (0.2 mL) intraperitoneally into each fish of the treatment group using a 1 mL syringe (HMD Global, Espoo, Finland). Similarly, control fish were injected with 0.2 mL of normal saline. During the experimental trial, the water temperature, pH, dissolved oxygen and alkalinity were followed as discussed above. Fish from both treated and control groups were sampled at an interval of 24, 48 and 72 h post-challenge for analysis of immune stress parameters. Briefly, the fishes were anesthetized with clove oil (Dabur, Ghaziabad, India) (50 µL per liter of water), and a 2 mL hypodermal syringe (24-gauge needles) were used to collect blood by puncturing the caudal vein of the fish. The collected blood samples in a 1.5 mL Eppendorf tube (without any anticoagulant) were stored in a refrigerator (4 °C) for 20–30 min. Afterward, the samples were centrifuged at 6000 RPM for 10 min at 4 °C and straw-colored supernatant serum was gently collected in sterile 1 mL centrifuge tubes and stored at −20 °C until further processing. All the procedures were carried out in sterilized conditions.

### 2.11. Immune-Stress Responses

The parameters like HSP70, HSP90, cortisol and IgM levels in serum were analyzed using commercial ELISA Assay Kit (BT BioAssay, Shanghai, China) and standard protocols following the manufacturer’s instruction. These kits were previously used [5] for the serum analysis in *L. rohita*. The assay was representative of four independent experiments, each performed in triplicate. In brief, 50 μL of the standard sample was added to a standard well containing a biotinylated antibody. Afterward, 40 μL of the sample, 10 μL anti-COR antibody, and 50 μL streptavidin-HRP were added to sample wells. The solution was thoroughly mixed and covered with a plate sealer. The plates were incubated at 37 °C for 60 min. The sealer was then removed and rinsed five times with a wash buffer. During each washing, a minimum of 0.35 mL of wash buffer was kept for 30 s to 1 min. Afterward, wells were filled with 50 μL of substrate solution A and 50 μL of substrate solution B. The plates were sealed and incubated for 10 min at 37 °C in dark conditions. Following that, 50 μL of stop solution was added to each well-containing sample and the color was observed to shift from blue to yellow. Within 10 min after adding the stop solution, each well’s optical density (OD) was measured using a microplate reader (BioTekEpoch^TM^2 Take-3 Plate Reader, Santa Clara, CA, USA) set to 450 nm.

### 2.12. Statistical Analysis

All data are presented as mean ± SE (standard error of the mean). The responses to serum immune stress parameters were subjected to one-way ANOVA followed by Tukey’s multiple comparison tests to identify significant differences. The level of significance was set at (*p* < 0.05). The statistical tests were performed in the SPSS statistical software (version 25).

## 3. Results

### 3.1. Biochemical Characterization

The isolated bacterial strain from moribund *L. rohita* displayed a Gram-negative character when subjected to Gram staining. The biochemical analysis showed that the isolated strain was positive for ONPG., urease, nitrate reduction, lysine utilization, citrate utilization, methyl red, indole, malonate utilization, saccharose, trehalose, glucose, caseinase, esterase, amylase and lecithinase. In contrast, the test showed negative for ornithine utilization, H_2_S production, phenylalanine deamination, Voges Proskauer’s, xylose, adonitol, esculin hydrolysis, arabinose, rhamnose, melibiose, cellobiose, lactose and raffinose. These results closely resemble the formerly reported biochemical test on the *A. veronii* strain. The comparative results are described in Table 2.

### 3.2. Morphological Characteristics by SEM Analysis

**Figure 2:**
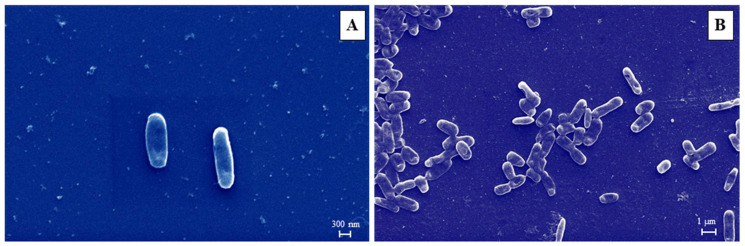
Scanning electron microscopy image of *A. veronii* bacteria (**A**) 25,000× magnification with scale bar (300 nm). (**B**) 10,000× magnification with scale bar (1 µm).

SEM analysis demonstrated that the phenotypic characteristics of the isolate are according to the morphological characteristics of *A. veronii*. The morphological feature of isolated *A. veronii* was observed at 25,000× and 10,000× (Figure 2). As the image shows, *A. veronii* displays a long, rod-shaped morphology with efficiency in producing biofilm. The scale bar has been projected in the picture.

### 3.3. Molecular Identification of Bacteria

**Figure 3:**
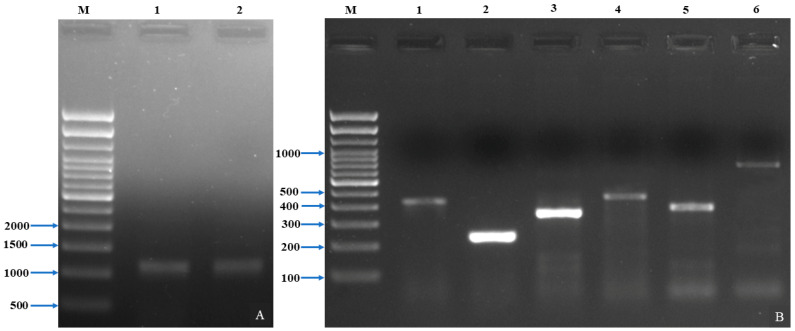
Gel image of amplification of 16S rRNA and virulent genes. (**A**) 16S rRNA gene (M-500 bp ladder, lane 1 and 2 amplicons of 1403 bp of 16S rRNA gene. (**B**) Amplicon of virulent genes (M-100 bp ladder, Lane 1-431 bp of *aerA*, Lane 2-232 bp of *act*, Lane 3-350 bp of *ser*, Lane 4-442 bp of *alt*, Lane 5-323 bp of *exu*, and Lane 6-891 bp of *ascV*.

**Figure 4:**
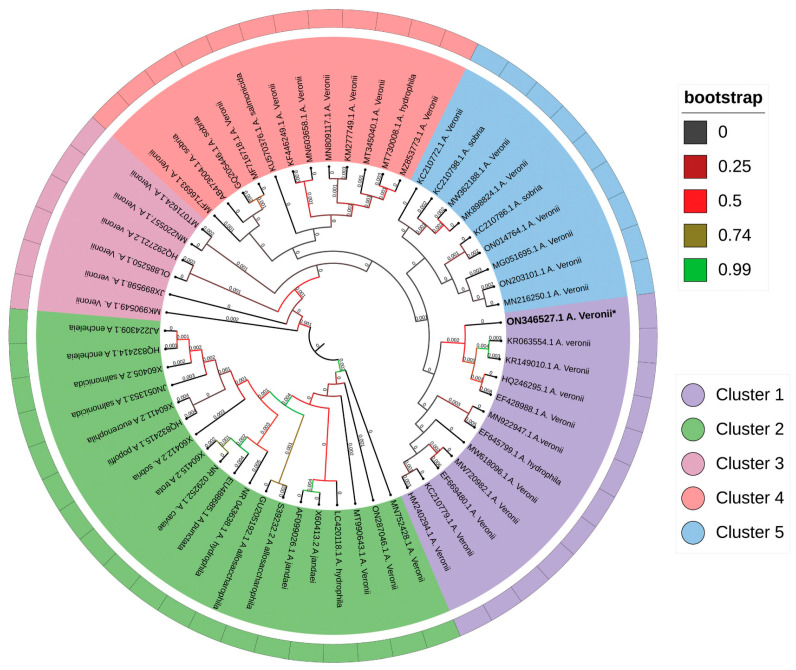
The phylogenetic tree preparation using iTOL V4 (Interactive Tree of Life) software. * Indicate the phylogenetic analysis of *Aeromonas veronii* based on 16S rRNA gene using the neighbor-joining method in MEGA 11.0. The number near the branches indicates the branch length up to three decimals. The legends next to the phylogeny show the bootstrap value for 1000 replications.

The 16S rRNA gene sequence analysis identified the isolated bacterial strain MP3 as *A. veronii*. The gene sequence was submitted to NCBI GenBank with Accession number ON346527. The gene sequence was subjected to a BLAST-N search, which revealed that the isolate was 99.43% identical with NCBI Gene Bank Accession Number MN603658 (isolate source: Tilapia, spleen) and MG051695 (isolate source: Rainbow trout, liver). The gel image of the 16S rRNA gene was screened (Figure 3A,B). The phylogenetic analysis revealed that the strain (MP3) was evolutionarily very close to other members of *Aeromonas* species (Figure 4).

### 3.4. Hemolysis Assay

**Figure 5:**
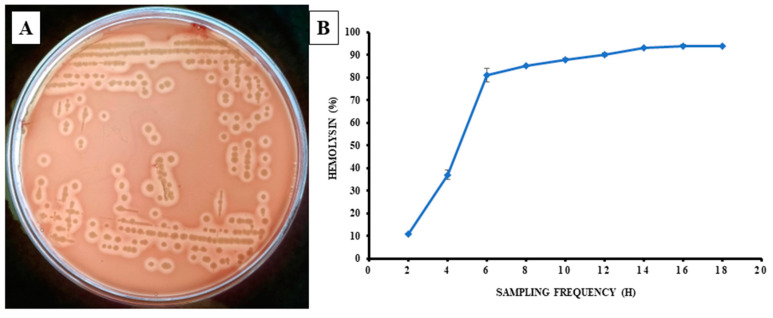
(**A**) β-hemolysin activity of *Aeromonas veronii* in blood agar supplemented with 5% sheep blood. (**B**) The percentage of hemolysis of sheep RBC produced by *A. veronii* was recorded at every 2 h interval until 18 h. All data are presented as mean ± S.E. (*n* = 6).

On the blood agar plate, the isolated bacterial strain demonstrated β-hemolysin activity, resulting in a clear zone surrounding the bacterium colony (Figure 5A). The hemolysis in the liquid phage experiment was examined every 2 h intervals for 18 h. After 18 h of incubation, the outcome indicated 94% hemolysis in sheep RBC at 16 h of post-incubation (Figure 5B). The bacteria were also streaked on Aeromonas-specific media and showed a visible colony after 24 h of incubation (Figure S2).

### 3.5. Antibiogram Study of A. veronii

An antibiogram assay revealed that the isolate MP3 was resistant to Dicloxacillin (D/C 1) and Ampicillin (AMP 25), whereas the strain was intermediate against Polymyxin B (PB300) and Imipenem (IPM10). However, the strain was found to be sensitive against Ofloxacin (OF2), Erythromycin (E 10), Gentamicin (GEN 10), Netilmicin sulfate (NET30), Amoxicillin (AMC 30), Tetracycline (TE10), Chloramphenicol (C30), Cefixime (CFM5), Piperacillin (PIT100/10), Nalidixic acid (NA30), Colistin (CL10), Doxycycline, (DO10), Trimethoprim (TR5), Fosfomycin (FO200), Rifampicin (RIF5), Nitrofurantoin (NIT 200), Tobramycin (TOB10), Cefepime (CPM30), Ciprofloxacin (CIP5), and Streptomycin (S25) (Table S1).

### 3.6. Occurrence of Virulence Genes

Among 11 virulence genes, the presence of genes *viz.*, *aerA*, *act*, *ser*, *alt*, *exu*, and *ascV* in *A. veronii* was confirmed through PCR (Table 3) and gel electrophoresis (Figure 3B). The results revealed that the identified virulent genes *aerA* was about 431 bp, *act* of 232 bp, *ser* of 350 bp, *alt* of 442 bp, *exu* of 323 bp, and *ascV* of 891 bp.

### 3.7. Determination of LD_50_

**Figure 6:**
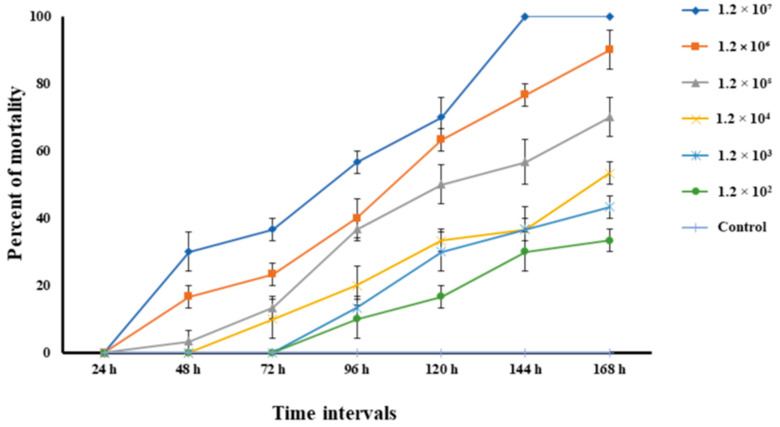
Cumulative mortality of challenged *L. rohita* fingerlings after intraperitoneal injection of pure culture of *A. veronii* at different concentrations. All data are presented as mean ± SE of three replicate tanks.

The cumulative mortality rates at different concentrations post-infection of *L. rohita* with *A. veronii* are shown in Figure 6. The control fish injected with sterile normal saline did not exhibit any mortality within seven days post-injection. The fish exposed to varying doses of intraperitoneal injection developed reddening and ulceration at the injection site. The bacteria were re-isolated from the blood, liver and kidney tissues and reconfirmed as *A. veronii* by sequencing the 16S rRNA gene. The LD_50_ value of *A. veronii* was calculated as 2.2 × 10^4^ CFU/fish when injected intraperitoneally.

### 3.8. Serum Biochemical Assay

**Figure 7:**
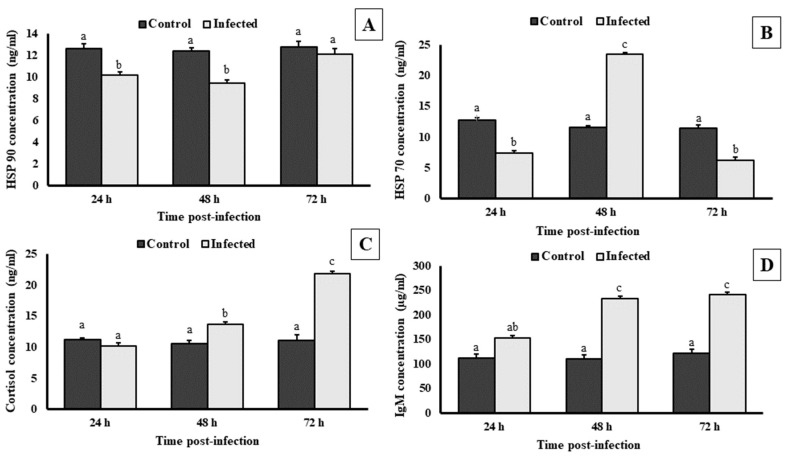
Immune-stress parameters of control and infected (bacteria injected) fingerlings of *L. rohita*. (**A**) represents the concentration level of HSP 90, (**B**) represents the concentration level of HSP 70, (**C**) represents fish cortisol concentration level and (**D**) represents the concentration level of IgM up to 72 h post-challenge. All data are presented as mean ± SE (*n* = 6), and different superscripts (c denotes highest value followed by b and a) indicate significant differences between treatment groups (*p* < 0.05).

After 24, 48 and 72 h post-challenge, the concentration of HSP 90, HSP 70, cortisol and IgM were not significantly (*p* < 0.05) changed in the control group serum samples. However, in the treatment group, HSP 90 activity was significantly (*p* < 0.05) decreased after 24 h and 48 h post-challenge as compared to the control. No significant (*p* < 0.05) changes in the concentration of HSP 90 were observed after 72 h post-challenge (Figure 7A). The HSP 70 activity showed a significant (*p* < 0.05) surge after 48 h of post-challenge when compared with the control group. However, the HSP 70 concentrations were significantly (*p* < 0.05) decreased after 24 and 72 h post-challenge (Figure 7B). Furthermore, the analysis revealed that the cortisol level was significantly (*p* < 0.05) increased after 48 and 72 h post-challenge as compared to the control group, whereas there was no significant (*p* < 0.05) difference observed after 24 h post-challenge (Figure 7C). The immunoglobulins (IgM) concentration was examined, and it was found that challenged fingerlings had significantly (*p* < 0.05) higher activity of IgM in serum after 24 h, 48 h, and 72 h post-challenge as compared to the control group (Figure 7D). The results revealed that both specific and non-specific immune system parameters of *L. rohita* were altered post-challenge of *A. veronii*.

## 4. Discussion

The Gram-negative *Aeromonas* species, ubiquitous in freshwaters, are opportunistic aquatic bacterial pathogens responsible for causing Aeromoniasis in fish species. For instance, *A. veronii*, *A. hydrophila*, *A. sorbia*, *A. jandaei*, *A. allosaccharophila* and *A. trota* render conventional Aeromoniasis in fish farming [15]. In the present study, *L. rohita* showed clinical signs on the skin surface and fins that resembled bacterial infections were collected, and isolated bacterial strain (MP3) was characterized as *A. veronii* based on biochemical analysis, morphological analysis by SEM, 16S rRNA gene sequencing and analysis of the phylogenetic tree. Similar observations on the isolation of bacterial species have been done previously based on clinical signs from oscar [25], Nile tilapia [48], catfish, and largemouth bass [24]. Pei et al. [17] revealed that the bacterial strain of *A. veronii* has a high virulent characteristic. The isolated strain in this study was streaked on the *Aeromonas*-specific medium and exhibited visible growth in the agar plate. Further, the strain induces significantly high mortality in healthy fingerlings of *L. rohita*.

Biochemical reactions can reveal the vital information necessary for accurately identifying the bacteria genera within a sample. By their nature, bacteria produce large volumes of enzymes, and it is these enzymes that allow for their identification via biochemical methods Kumar et al. [50]. For instance, Zhao et al. [51] highlighted the importance of biochemical characterization in bacterial identification. In the study, a total of 36 bacterial isolates recovered from samples collected at Nanchang City, Jiangxi Province, China, were identified through specific enzymatic activity. Syed et al. [52] demonstrated that a newly isolated strain can be identified as *Enterobacter* sp. based on variable biochemical reactions. Hence, the type of enzymes produced by a bacterium can usually be used to classify its species, given that bacteria have distinct enzymatic profiles. In the present study, the recovered *A. veronii* showed positive activity for citrate utilization, indole, trehalose, glucose, caseinase, esterase, amylase, and lecithinase. In contrast, negative activity was reported for esculin hydrolysis, xylose, cellobiose, melibiose, raffinose, and lactose. The results were in parallel with previous findings where pathogenic *A. veronii* demonstrated a similar pattern in substrate utilization, especially for hemolysin, caseinase, esterase, amylase and lecithinase [17,31,49]. Our results suggest that isolated *A. veronii* is potentially a highly lethal bacterial pathogen utilizing various substrates for energy and growth. However, the substrate of utilization could vary between different pathogenic isolates recovered from distinct geographical regions; hence, further in vivo assays, like survival, hemolysin, virulent gene presence, etc., must be carried out to confirm the pathogenicity of isolated strains.

The morphological analysis illustrates the structure of this *A. veronii* rod-shaped, and the average length and width varied from 1.0–3.5 µm and 0.3–3.5 µm, respectively [53,54]. A similar observation in this study was noticed by SEM analysis of the isolated bacterial strain (MP3) from *L. rohita* with the character of producing biofilm. The phylogenetic analysis of the 16S rRNA gene sequence of the MP3 strain revealed that the isolate was 99.43% identical with MN603658 (isolate source: Tilapia, spleen) and MG051695 (isolate source: Rainbow trout, liver). The sequences were clustered with the highest bootstrap values in the phylogenetic tree. The pathogenic strains of *A. veronii* were previously isolated from different diseased fishes and confirmed by 16S rRNA gene sequencing [17,18,23].

This study highlighted that the MP3 strain of *A. veronii* showed high β-hemolytic phenotypic growth on the blood agar supplemented with sheep blood, which indicated its pathogenicity towards fish species. Similarly, the *A. veronii* isolated from diseased *Anabas testudineus* showed β-hemolytic activity that has been reported previously [19]. The wide use of antibiotic doses resulted in antimicrobial resistance to pathogenic bacteria [55]. The Aeromonas are genetically resistant to ampicillin [8]. In the present study, the isolated strain (MP3) from *L. rohita* showed resistance against Ampicillin and Dicloxacillin. The same bacteria isolated from *Micropterus salmoides* [17] showed resistance against Norfloxacin, Tetracycline, Doxycycline, Kanamycin Ampicillin, Penicillin G, Nalidixic acid, Trimethoprim-sulfamethoxazole. Because various strains of bacteria have variable drug susceptibility, the efficiency of antibiotics used to combat the bacteria should be verified before use in clinical practice [56].

The (*aerA*) codes for the aerolysin gene responsible for releasing toxins that form pores on the epithelial cells leading to damage to the cells [57]. Similarly, the gene (*act*) codes for cytotoxic enterotoxin, which decreases the capacity to induce fluid secretion in the intestine [58]. Other genes like heat-labile cytotonic enterotoxin (*alt*) and serine protease (*ser*) are the enzymes that are responsible for the pathogenicity of *Aeromonas* infection [59]. Similarly, the *alt* gene accountable for producing cytotoxin and enterotoxin is crucial in establishing the infection [60]. Dnase (*Exu*) production primarily depends on the nutritional requirement of the pathogen [61]. The production of the Dnase protein leads to the breakdown of DNA. in the organism, which leads to pathogenesis [46,62]. Type III secretion is vital in pathogenicity because it directly facilitates toxins entering the host system [63,64,65,66]. In contrast, virulent genes including *gcaT* (*Glycerophospholipid: cholesterol acyltransferase*), *Lip* (*Lipase*), *ast* (*Cytotonic enterotoxin*), *ahyB* (*Elastase*) and *hlyA* (*Hemolysins*) were absent in the isolated *A. veronii* strain. This highlights the dynamics of virulent gene presence in *A. veronii.* It might be possible that *A. veronii* doesn’t require the expression of all the virulence genes to induce high toxicity in fish. However, it needs further validation. Moreover, the pathogenicity of the isolated strain (MP3) was verified by the challenge study through intraperitoneal injection of pure culture (MP3 strain) in fingerlings of healthy *L. rohita*. The LD_50_ of the isolated *A. veronii* strain was 2.2 × 10^4^ CFU/fish. In contrast, LD_50_ in *M. salmoides* infected by *A. veronii* was reported as 3.72 × 10^4^ CFU/fish [17]. Similarly, the value of LD_50_ in *Carassius auratus* was 1.31 × 10^7^ [18].

Small heat shock protein (HSP) groups like HSP90, HSP70, HSP60, HSP40, and HSP110 play a crucial role in dealing with environmental stress, such as preventing protein damage when tissues are exposed to high heat and chaperoning DNA repairs as part of the primary stress response associated with disease conditions [67]. In this study, we analyze the concentration level of HSP90 and HSP70 from the serum samples of control and bacterial-challenged fishes. The serum analysis showed a change in both HSP 70 and HSP 90 levels after different points in time. There is a link between reduced stress and increased antioxidative response and non-specific (HSP70 and HSP90) and specific immunological response (IgM) of animals to microbial infection [68]. Interestingly, our result showed that the increase in non-specific immune response triggers the specific immune response like IgM in the fish challenged with the pathogenic *A. veronii* strain. The stress response is an adaptation that helps fish deal with actual or potential threats to maintaining their normal or homeostatic state [69]. Cortisol is released in response to chronic and acute stress, increasing energy expenditure and somatic energy expenditure storage [5]. Cortisol levels in the blood are widely used to assess the stress level experienced by fish [70,71]. In accordance with the present study, the cortisol level in *L. rohita* elevated from 24 h post-challenge. Similarly, the fish *L. rohita* challenge with other species of *Aeromonas* (*A. hydrophila)* positively correlated with an elevated cortisol level of 24 h post-challenge [72].

## 5. Conclusions

The present study isolated a pathogenic bacteria strain from farmed *L. rohita* in West Bengal, India. Based on the biochemical analysis, SEM analysis and 16S rRNA gene sequencing, the MP3 strain was identified as *A. veronii.* The strain has significant toxin accessory genes like *aerA*, *act*, *ser*, *Lip*, *alt*, *exu*, and *ascV*, which collectively contribute to the virulence of the bacterium. The in vivo challenge study highlighted that *A. veronii* induces significant cellular changes, resulting in the high mortality rate of *L. rohita*. Furthermore, the isolated strain was sensitive against most tested antibiotics except Ampicillin and Dicloxacillin. The results of serum analysis revealed changes in the concentration of cortisol, HSP 70, HSP 90 and IgM levels. Together, our results add new information about *A. veronii* infection reported in *L. rohita* and advance our knowledge of various characteristics of this bacterium. Further research through RNA-seq analysis will improve the understanding of the immune mechanisms of IMC (e.g., *L. rohita*) in response to *A. veronii* infection. Additionally, information on significant genes involved in immune responses will be generated, which would aid in the development of marker genes to develop therapeutics and help to manage diseases caused by this pathogen in the aquaculture system.

## Figures and Tables

**Table 2:** Comparison of the biochemical test result of *A. veronii* isolated from *L. rohita*.

Sl. No	Test	ON346527	Pei. et al., 2021 [17]	Dong et al., 2017 [48]	Abott et al., 2003 [49]
1	ONPG	+	ND	+	+
2	Urease	+	ND	−	−
3	Nitrate reduction	+	ND	ND	ND
4	Citrate utilization	+	+	ND	+
5	Methyl red	+	ND	+	ND
6	Indole	+	+	+	+
7	Malonate utilization	+	ND	ND	−
8	Saccharose	+	ND	+	ND
9	Trehalose	+	+	ND	ND
10	Glucose	+	+	+	+
11	Lysine utilization	+	ND	+	ND
12	Ornithine utilization	−	N.D.	−	ND
13	Phenylalanine deamination	−	ND	ND	ND
14	H_2_S production	−	ND	ND	ND
15	Voges Proskauer’s	−	+	−	ND
16	Esculin hydrolysis	−	−	ND	−
17	Arabinose	−	N.D.	−	ND
18	Xylose	−	−	ND	ND
19	Adonitol	−	ND	ND	−
20	Rhamnose	−	N.D.	−	−
21	Cellobiose	−	−	ND	+
22	Melibiose	−	−	−	−
23	Raffinose	−	−	ND	−
24	lactose	−	−	ND	−
25	Oxidase	+	+	ND	ND
26	Caseinase	+	+	+	+
27	Esterase	+	+	+	+
28	Amylase	+	+	+	+
29	Lecithinase	+	+	+	+

Values in positive (+), negative (−) and ND (not done).

**Table 3:** The presence of virulence genes detected through PCR amplification.

Protein Product	Target Gene	Amplicon Size	Detection
Aerolysin	*aerA*	431	+
Cytotoxic enterotoxin	*ac*	232	+
serine protease	*ser*	350	+
Glycerophospholipid: cholesterol acyltransferase	*gcaT*	237	−
Lipase	*Lip*	382	−
Cytotonic enterotoxin	*ast*	331	−
Heat-labile cytotonic enterotoxin	*alt*	442	+
Elastase	*ahyB*	513	−
DNase	*exu*	323	+
Hemolysins	*hlyA*	597	−
Type III Secretion System	*ascV*	891	+

Presence or absence of vierulent genes is indicated by (+) and (−).

## Data Availability

Raw 16S rRNA sequencing data of *Aeromonas veronii* have been deposited to the NCBI under accession number ON346527.

## Supplementary Materials

The following supporting information can be downloaded at: https://www.mdpi.com/article/10.3390/pathogens12040598/s1. Figure S1. Map showing sampling location of infected *L. rohita* at Purba Medinipur, West Bengal, India; Figure S2. Appearance of *A. veronii* colony on *Aeromonas* specific agar media; Table S1. Antibiotics susceptibilities of *A. veronii* isolated from diseased *Labeo rohita*.
