# Supplementary material for: Aeromonas veronii Is a Lethal Pathogen Isolated from Gut of Infected Labeo rohita: Molecular Insight to Understand the Bacterial Virulence and Its Induced Host Immunity
Source: Pathogens. 2023 Apr 14;12(4):598. doi: 10.3390/pathogens12040598 (PMC10143776; doi:10.3390/pathogens12040598)
Supplement: Supplementary file 1 [file pathogens-12-00598-s001.zip › pathogens-2305449-supplementary.pdf]

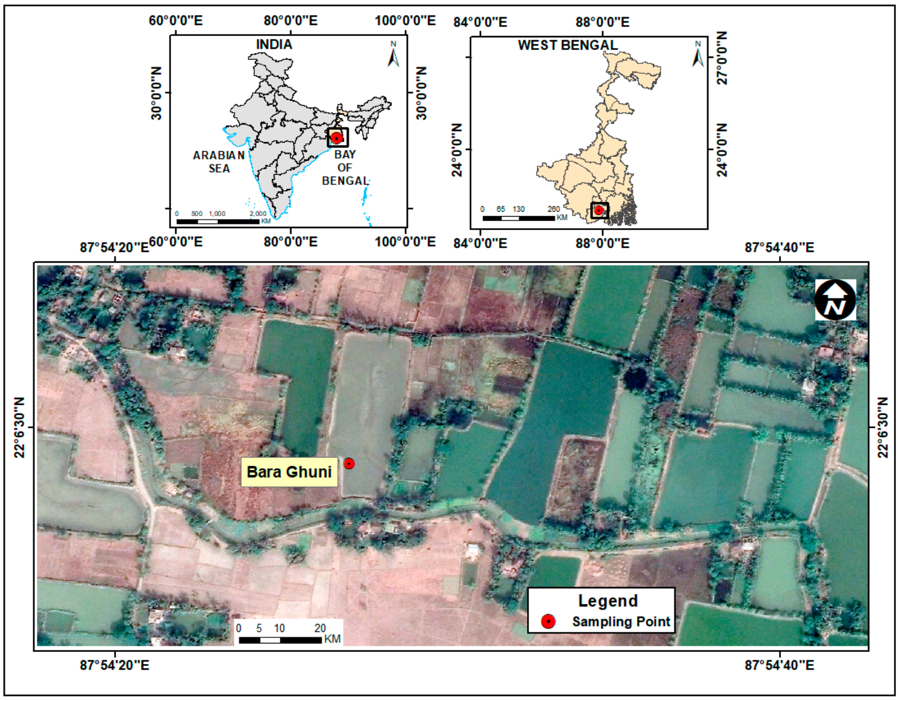

Figure S1. Map showing sampling location of infected *L. rohita* at Purba Medinipur, West Bengal, India.

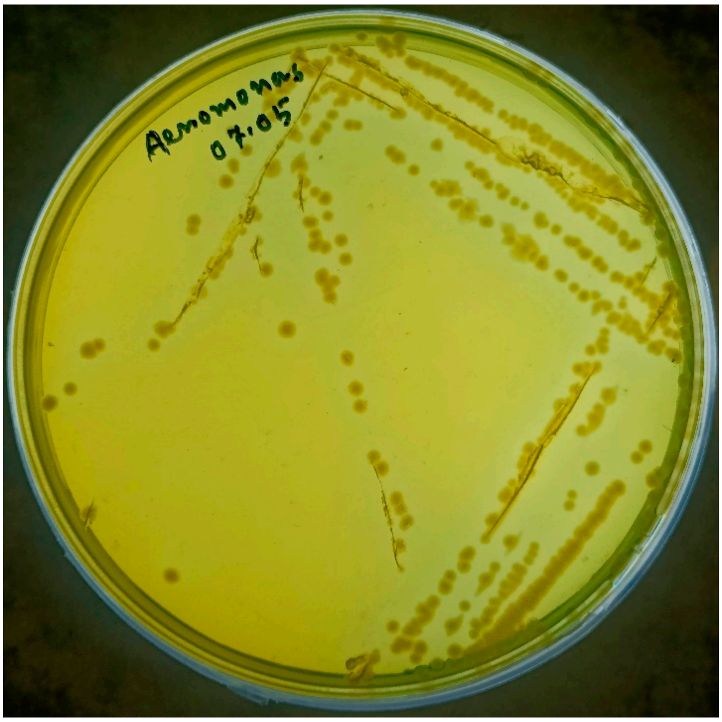

Figure S2. Appearance of *A. veronii* colony on *Aeromonas* specific agar media.

Table S1. Antibiotics susceptibilities of *A. veronii* isolated from diseased *Labeo rohita.*

Sl. No.	Antimicrobial	Disc concentration (µg)	Mean zone diameter (mm)	Sensitivity
1.	Ampicillin	25	0	R
2.	Dicloxacillin	1	0	R
3.	Ofloxacin	2	25	S
4.	Erythromycin	10	34	S
5.	Gentamicin	10	28	S
6.	Netilmicin sulphate	30	45	S
7.	Amoxicillin	30	22	S
8.	Tetracycline	10	25	S
9.	Chloramphenicol	30	31	S
10.	Cefixime	5	35	S
11.	Piperacillin	10	39	S
12.	Nalidixic acid	30	28	S
13.	Imipenem	10	18	I
14.	Colistin	10	32	S
15.	Doxycycline	10	39	S
16.	Trimethoprim	5	28	S
17.	Fosfomycin	200	41	S
18.	Rifampicin	5	35	S
19.	Nitrofurantoin	200	24	S
20.	Tobramycin	10	36	S
21.	Cefepime	30	26	S
22.	Polymyxin B	300	12	I
23.	Ciprofloxacin	5	26	S
24.	Streptomycin	25	36	S

Note: R denotes resistant, S denotes Sensitive and I denote Intermediate
